# Correlation between the Serum Concentration of Vitamin A and Disease Severity in Patients Carrying p.G90D in *RHO*, the Most Frequent Gene Associated with Dominant Retinitis Pigmentosa: Implications for Therapy with Vitamin A

**DOI:** 10.3390/ijms24010780

**Published:** 2023-01-02

**Authors:** Tjaša Krašovec, Nina Kobal, Maja Šuštar Habjan, Marija Volk, Marko Hawlina, Ana Fakin

**Affiliations:** 1 Eye Hospital, University Medical Centre Ljubljana, Grablovičeva ulica 46, 1000 Ljubljana, Slovenia; 2 Clinical Institute of Medical Genetics, University Medical Centre Ljubljana, Šlajmajerjeva ulica 4, 1000 Ljubljana, Slovenia; 3 Faculty of Medicine, University of Ljubljana, Vrazov trg 2, 1000 Ljubljana, Slovenia

**Keywords:** retinitis pigmentosa, RP, vitamin A, CSNB, NBWD, congenital stationary night blindness, rhodopsin, RHO, sector RP, treatment

## Abstract

The pathogenic variant p.G90D in *RHO* is believed to be responsible for a spectrum of phenotypes, including congenital stationary blindness (for the purpose of this study termed night blindness without degeneration; NBWD), Sector RP, Pericentral RP, and Classic RP. We present a correlation between the serum concentration of vitamin A and disease severity in patients with this variant. This prospective study involved 30 patients from 7 families (17 male; median age 46 years, range 8–73). Full ophthalmological examination including visual acuity, Goldmann perimetry, slit-lamp exam, optical coherence tomography, fundus autofluorescence, and electrophysiology was performed to determine the presenting phenotype. The serum concentration of vitamin A was determined from a fasting blood sample taken on the day of the exam, where it was found that 23.3% (7/30) of patients had NBWD, 13.3% (4/30) had Sector RP, 3.3% (1/30) had Pericentral RP, and 60% (18/30) had Classic RP. Multiple logistic regression revealed a significantly higher probability of having a milder phenotype (NBWD or Sector RP) in association with younger age (*p* < 0.05) and a higher concentration of vitamin A (*p* < 0.05). We hypothesize that vitamin A in its 11-*cis*-retinal form plays a role in stabilizing the constitutively active p.G90D rhodopsin and its supplementation could be a potential treatment strategy for p.G90D *RHO* patients.

## 1. Introduction

Retinitis pigmentosa (RP) is a group of genetically different diseases, that together represent the most frequent inherited retinal disease, affecting 1 in 4000 people worldwide [1]. With the exception of one genetic subtype (*RPE65*) [2], the disease is as of yet untreatable and leads to blindness in the majority of patients [1]. Vitamin A, one of the main molecules involved in visual transduction, has been repeatedly considered as a possible therapy [3,4,5,6,7]; however, its therapeutic value has been disputed [8] and it has not been accepted as a mainstay therapy for RP.

Pathogenic variants in the rhodopsin gene (*RHO*) are the most frequent cause of dominant RP [1,9,10,11,12]. The encoded protein (rhodopsin) is a G-protein coupled receptor (GCPR) and a key light-sensitive protein of rod photoreceptors, which directly bind 11-*cis*-retinal, the active compound of vitamin A [13], together forming a rhodopsin molecule [14]. Besides classic RP, other phenotypes have been associated with *RHO,* including sector RP [15], pericentral RP [16], and congenital stationary night blindness (CSNB) [9,10,11,12,13,15]. The reason for the phenotypic variability is not clear and has been mostly attributed to differences between genetic variants. However, recently, all four phenotypes were observed in patients with an identical *RHO* variant, p.G90D [17], suggesting the existence of additional factors affecting the phenotype, external to the causative variant. The purpose of this study was to determine whether there was a correlation between the serum concentration of vitamin A and the phenotypes in these patients.

## 2. Results

**Figure 1:**
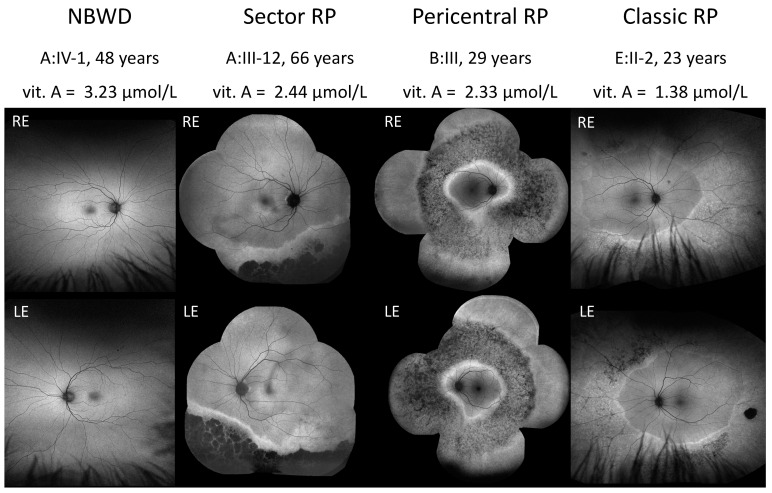
Representative cases from each phenotypic group. NBWD—night blindness without degeneration; RP—retinitis pigmentosa; vit. A—serum concentration of vitamin A; RE —right eye; LE—left eye.

The phenotypes of the patients were classified into four categories: (1) Night blindness without degeneration (NBWD) (7/30, 23.3%), (2) Sector RP (4/30, 13.3%), (3) Pericentral RP (1/30, 3.3%), and (4) Classic RP (18/30, 60%). Figure 1 shows the fundus autofluorescence images (FAF) of the representative cases from each phenotypic group.

A summary of the patients’ clinical characteristics and their serum concentrations of vitamin A are shown in Table 1. Approximately half of the patients (N = 14) had been described in detail in a previous publication [17].

### 2.1. Correlation between Serum Vitamin A Concentration and Age

**Figure 2:**
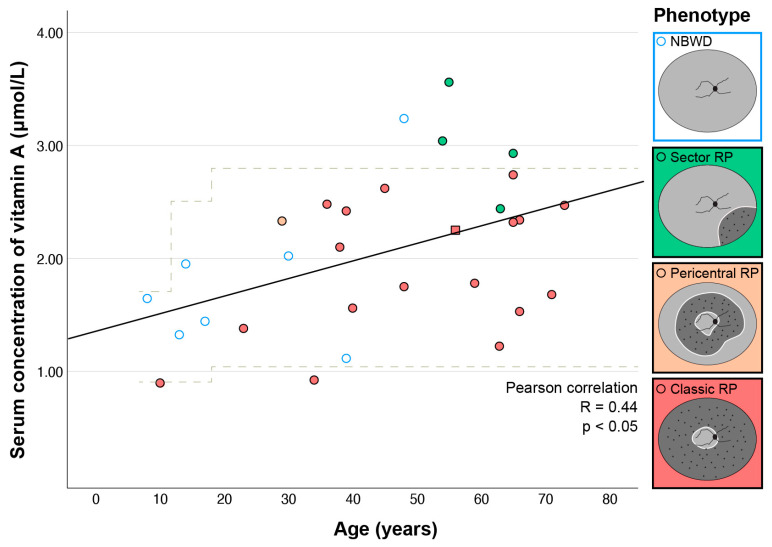
Correlation between serum vitamin A concentration and age. Different phenotypes are marked with different colors. The patient taking vitamin A supplements is marked with a square. Most patients with NBWD were younger than 40 years. Note that their serum concentration is relatively low (most likely due to younger age), but it is mostly higher than that of RP patients of similar ages. In patients aged ≥40 years, higher vitamin A concentrations were found in patients with a mild phenotype (NBWD or Sector RP) than in patients with a severe phenotype (Classic RP). The laboratory reference values were 0.91–1.71 µmol/L (7–12 years old), 0.91–2.51 µmol/L (13–18 years old), and 1.05–2.8 µmol/L (adults); marked with dashed lines. NBWD—night blindness without degeneration; RP—retinitis pigmentosa.

Here, 80% of patients (24/30) had a serum vitamin A concentration within the normal range for their age, 7% (2/30) below it, and 13% (4/30) above it. To check whether the measured concentration of serum vitamin A was reliable; the measurement was repeated in two patients one year after first examination (A:III-11 and A:III-14). The first and second value were 2.79 vs. 2.93 for patient A:III-11 and 3.56 vs. 3.56 for patient A:III-14, respectively. The two with concentrations below normal both had classic RP. Among those with concentrations above normal, three of them had sector RP and one had NBWD. One patient (Patient G: I-1) had a history of taking vitamin A supplementation, and her concentration was 2.25 (normal range 1.05–2.8 for her age). She was excluded from further statistical analysis due to the exogenous intake of vitamin A. There was a significant correlation between vitamin A concentration and age (Figure 2) (Pearson correlation, R = 0.44, *p* < 0.05). 

### 2.2. Correlation between Serum Vitamin A Concentration and Phenotype

**Figure 3:**
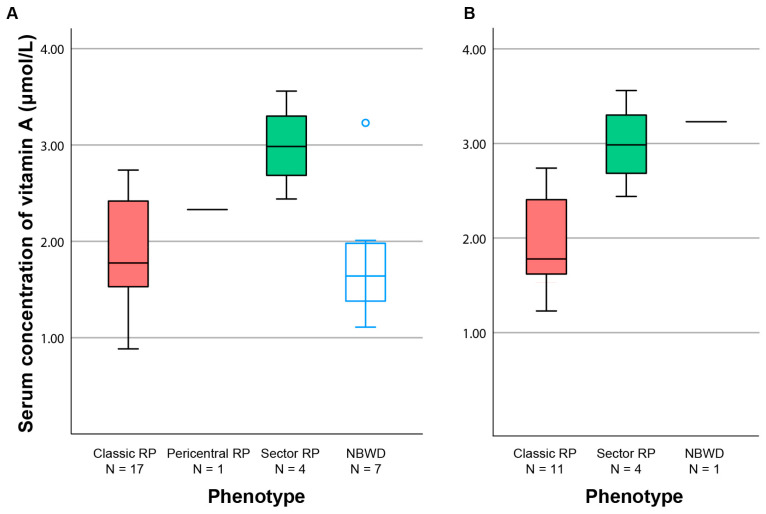
Boxplot chart showing the serum vitamin A concentration according to the phenotype. Boxes include half of the data and whiskers show the remaining data. The horizontal line indicates the median value (or the value of one patient with pericentral RP). (**A**) The whole cohort. (**B**) Patients aged ≥40 years. Note only one remaining patient with NBWD. RP—retinitis pigmentosa; NBWD—night blindness without degeneration.

All patients, except one with NBWD, were younger than 40 years (see Figure 2), suggesting that NBWD was not a stationary phenotype, but rather a disease stage preceding retinal degeneration. In the subgroup of patients aged ≥40 years (N = 16), in whom we presumed that the progressed clinical picture had likely already developed, there were significant differences in the median vitamin A concentrations between patients with different phenotypes (Kruskal–Wallis test, *p* < 0.05). A pairwise comparison showed significant difference between Sector RP and Classic RP (2.99 vs. 1.78 µmol/L, *p* < 0.05) (Figure 3B). When patients were grouped into mild (Sector RP or NBWD) and severe (Classic RP) phenotypes, the significance increased (median 3.04 vs. 1.78 Mann–Whitney test, *p* < 0.01). When looking at the entire cohort, the differences between the four groups were statistically significant (Kruskal–Wallis test, *p* < 0.05), but the pairwise comparisons were not significant (Figure 3A).

As age affects vitamin A concentration (see above) and disease stage (considering that RP is a progressive disease), multiple logistic analysis was performed to determine the association between the phenotype and serum concentration of vitamin A when accounting for the effect of age. For this analysis, phenotypes were grouped in two groups, mild (NBWD, Sector RP) and severe (Classic RP and Pericentral RP), to increase statistical power. Sector RP was considered mild as it has been described previously as being mostly stationary [18,19]. Pericentral RP was considered severe as it affects a larger part of the functional vision, and the patient had notably reduced electroretinography (ERG) amplitudes (see Figure 3 in Kobal et al.) [17]. Multiple logistic regression revealed a significantly higher probability of having milder phenotype (NBWD or Sector RP) in association with younger age (*p* < 0.05) and a higher concentration of vitamin A (*p* < 0.05) (details in Table 2). The association is further demonstrated in Figure 2.

**Figure 4:**
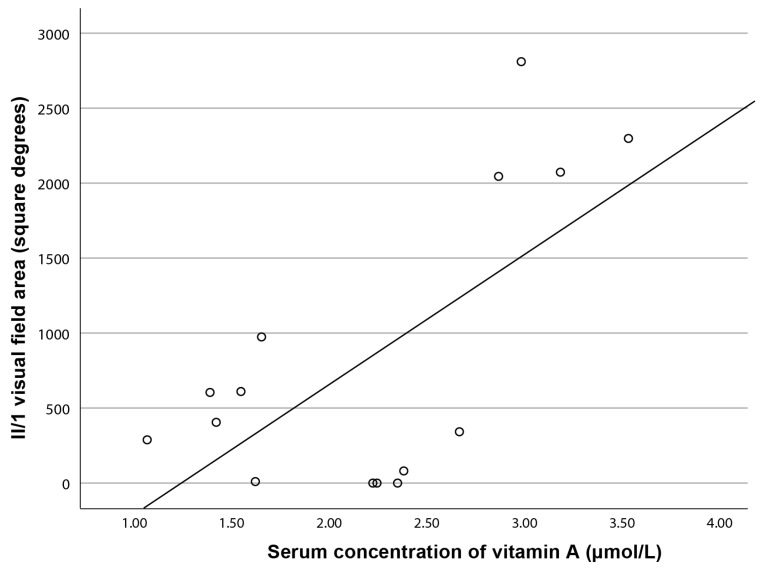
Correlation between the visual field area (isopter II/1) and serum concentration of vitamin A in patients aged ≥40 years.

In the subgroup of patients aged ≥40 years (N = 16), there was a significant correlation between the serum vitamin A concentration and visual field area for isopter II/1 (Pearson correlation, R = 0.66, *p* < 0.01), but not for II/4 (R = 0.50, *p* = 0.059) (Figure 4). Including the entire cohort, there was no statistically significant correlation between the serum vitamin A concentration and visual field area. There was no statistically significant correlation between the serum vitamin A and ERG amplitudes.

## 3. Discussion

This study is, to the best of our knowledge, the first to date to provide evidence of a correlation between a higher serum concentration of vitamin A and a milder phenotype in patients with *RHO* retinopathy.

### 3.1. Correlation between Serum Concentration of Vitamin A and Age

There was a significant correlation between the serum vitamin A concentration and age, with higher vitamin A concentrations observed at older ages (Figure 2). This is in accordance with the literature [20,21,22] and is reflected in different normative levels for different age groups. Vitamin A is absorbed from food in the form of retinyl ester and transported to the liver through the lymphatic system [20]. After uptake in the liver, retinyl esters are stored in stellate (Ito) cells. The liver is the main storage site of vitamin A and contains 80% of total body reserves [23]. The increase in serum concentration of vitamin A (retinol) with age may linked to higher liver reserves. Other storage sites include retinal pigment epithelium (RPE) and adipose tissue and lungs [24,25,26,27]. After their uptake in the liver, retinyl esters are hydrolyzed into retinol, which forms the complex (holo-RBP) with retinol binding protein (RBPP4), the main vitamin A carrier. The complex is transported to the peripheral tissues [20,27,28,29,30]. In the choroid, it diffuses from choroidal blood circulation through the Bruch’s membrane to the RPE cells, where it binds to the RBP4 receptors (stimulated by retinoic acid 6, STRA6) [27,31,32]. After binding to STRA6, all-*trans*-retinol is transported to the cytoplasm and bound to cellular retinol-binding protein 1 (CRBP1) [27,33,34,35]. CRBP1 transfers it to the enzyme retinol: lecithin acyltransferase (LRAT), which converts it to all-*trans*-retinyl esters [27,36,37]. They are substrates for retinoid isomerohydrolase (RPE65) that catalyze their isomersation to 11-*cis*-retinol, which is then bound by a cellular retinaldehyde-binding protein (CRALBP) and oxidated to 11-*cis*-retinal [24,27,38,39]. It is transferred into the interphotoreceptor matrix and, after being bound to interphotoreceptor retinoid-binding protein (IRBP), is delivered to the outer segments of the photoreceptors where it forms a visual pigment after binding with opsin [27,36,40].

### 3.2. Correlation between Serum Concentration of Vitamin A and Phenotype

The study demonstrated a significant correlation between a higher serum concentration of vitamin A and a milder phenotype of patients with *RHO*-retinopathy (Table 2). 

The analysis had to be performed in consideration of patients’ age, as age can affect both the serum concentration of vitamin A and the phenotype. 

Observing the data, among patients aged ≥40 years, in whom we presume the progressed clinical picture had likely already developed (see Figure 2), only one patient (1/17; 6%) had no retinal degeneration (NBWD), where this was observed in 46% (6/13) of patients younger than 40 years. It is very likely that in *RHO* p.G90D carriers, NBWD is, in most cases, not a distinct phenotype, but rather a phenotype preceding RP (classic, pericentral or sector type). This is in accordance with the observations of Sandberg et al., who found that in *RHO*-RP, visual deterioration usually occurs after the age of 40 [41]. Considering this, we analyzed the correlation between vitamin A and the phenotype primarily in patients aged ≥40 years. In this group, the patients with classic RP had notably lower (but still within normal limits) vitamin A concentrations than patients with sector RP and NBWD, whose vitamin A concentration was in 4/5 cases above the normal level (Figure 2). The difference in vitamin A concentration between the patients with severe retinal disease (classic RP) and mild disease (NBWD or sector RP) was significant, and strongly suggests that the vitamin A level plays a role in the development of retinal degeneration in patients harboring p.G90D in *RHO*. This was further supported by multiple logistic regression that included the whole patient group and accounted for the effect of age, which confirmed a significantly increased risk of severe retinal degeneration in patients with a lower serum concentration of vitamin A. It is possible that our patients, who are younger than 40 years, do not yet have a fully developed degeneration, and that in the case of NBWD it will still appear; however, longitudinal research is needed for definitive confirmation. 

A study performed by Campbell et al. several decades ago included 71 RP patients and showed abnormally low vitamin A concentrations in 91% patients [21]. After treatment with supplementation, the research group noted an increased vitamin A concentration and signs of slower disease progression [42]. Although the patients were not genetically confirmed, several had a family history, suggesting genetic etiology at least in this subgroup. While that RP cohort had abnormally low vitamin A levels (38–162 IU/100 mL (normal range 69–157 IU/100 mL)) [21], the RP cohort in this study had normal vitamin A levels and the Sector RP had abnormally high levels. It is possible that these differences are a reflection of a different laboratory normative. Another possibility is that patients with milder disease exhibit a difference in vitamin A metabolism or the availability of vitamin A from liver stores. 

### 3.3. The Use of Vitamin A as a Treatment Strategy

Besides Campbell et al. [42], the use of vitamin A as a treatment for RP has been studied mostly by the research group of Berson et al., who conducted a number of studies on the efficacy and safety of vitamin A supplementation in RP patients [3,4,5,6,7,20]. They were able to demonstrate that supplementing with 15,000 IU of vitamin A in the form of retinyl palmitate daily statistically significantly reduced the annual loss of residual ERG amplitude of cones. On the other hand, they did not observe an effect on slower narrowing of visual field compared to the placebo [3,4,5]. The authors suggest that it was possible that some patients with this disease have a reduced ability to retain vitamin A in the retina due to defective rods and cones. As another possibility, it may be as a result of the disturbed transport of vitamin A from the serum to the retina [3]. Despite the research mentioned above, the use of vitamin A in the treatment of RP has not entered widespread clinical use. Schwartz et al. [8] concluded that, based on the results of the randomized trials to date, there is no clear evidence of a benefit from the treatment of RP with vitamin A in terms of the mean change in visual field area or ERG amplitudes after one year of treatment and a mean change in visual acuity at the five-year follow-up. In addition, exclusion criteria in the summarized trials were extensive, excluding patients with atypical forms of RP (e.g., Sector RP), people aged over 55 years, patients with severe disease, pregnant women, patients with hepatic impairment, and patients with weight and height below fifth percentile [8]. Other disadvantages of their studies were that the serum vitamin A concentrations were not measured and the patients were not genotyped.

In the present study, there was a correlation between milder phenotypic groups and the serum concentration of vitamin A, a modest correlation between visual field area and serum concentration of vitamin A, and no correlation between the ERG amplitude and serum concentration of vitamin A. It is most likely that the statistical analysis was not able to show correlations for visual field and ERG amplitudes due to the strong confounding factors of the progressive nature of disease and the increase in vitamin A with age. Visual field area and ERG amplitudes normally decrease with age in patients with retinal dystrophies, which, together with the normal increase in vitamin A with age, results in generally worse parameters at higher vitamin A levels. As our patients were of different ages, it was thus very difficult to show the protective effect of vitamin A. Careful statistical analysis considering all confounding factors on a large patient group is needed to definitely show to what extent serum vitamin A plays a role in the *RHO* phenotype.

It is important to note that patients whose underlying disease-causing gene has not been identified, or who have an ABCA4 mutation, likely should not generally supplement with vitamin A.

### 3.4. Possible Role of Vitamin A in Retinal Degeneration in RHO p.G90D Carriers

Vitamin A is the name given to a group of different molecules with a similar chemical structure. The most important forms of vitamin A include retinoic acid and retinal [43]. The latter is key to visual perception. For visual perception (transduction) that occurs in rod photoreceptors, 11-*cis*-retinal needs to be bound to opsin, which together form the visual pigment, rhodopsin [14]. Upon the absorption of a photon, 11-*cis*-retinal is isomerized to all-*trans*-retinal, activating the opsin molecule [14]. For vision, it is important that all-*trans*-retinal molecules are regenerated to their initial state (11-*cis*-retinal), because otherwise both rods and cones would eventually remain without visible pigment. The process of regeneration is called the visual cycle, the retinoid cycle, or the regeneration cycle. The regeneration cycle in rods occurs during dark adaptation [14].

Retinoids, a group of chemical compounds that are derivates of vitamin A, are, in addition to their role in visual transduction in photoreceptors, also necessary for their survival [43]. 

Normal rod function and appropriate adaptation to darkness depend on the appropriate ratio between rhodopsin synthesis and breakdown. In addition, this process also requires retinal reductase, which uses vitamin A for activation [43]. A clue to the role of vitamin A in development of RP is the mechanism in which retinal deterioration occurs in vitamin A deficiency in people without genetic defects. In vitamin A deficiency, the earliest sign is dysfunction of the rods and thus the deterioration of night vision. The reason for this is the lack of 11-*cis*-retinal for the regeneration of the visual pigment, but also the inability to eliminate active forms of opsin [14]. In long-term deficiency, structural changes in the photoreceptors may be seen [27,44,45,46,47] and even severe degeneration [14]. The cause of the degeneration is thought to be related to multiple factors, including the toxicity of the constant activation of transduction, which occurs even in the absence of the chromophore, as well as the misfolded proteins that occur due to the absence of the chromophore during the opsin gene translation itself [14]. 

**Figure 5:**
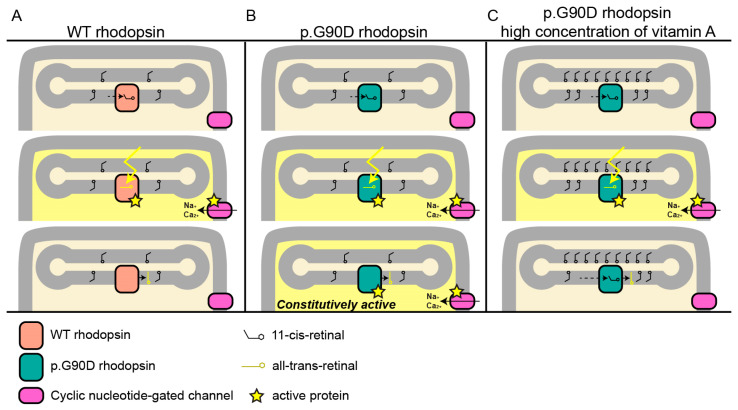
Proposed mechanism in which a higher concentration of vitamin A reduces the pathogenic effect of p.G90D. (**A**) Normal visual transduction. Light triggers the change in conformation from 11-*cis*-retinal to all-*trans*-retinal, which activates rhodopsin, which, in turn, via a series of events (not shown) causes the cyclic nucleotide-gated channel to open, allowing for the influx of Na^+^ and Ca^2+^. (**B**) Constitutively active p.G90D rhodopsin. (**C**) Hypothesized reduction in the activity of p.G90D rhodopsin by increasing the concentration of vitamin A, and with that of 11-*cis*-retinal, which inactivates the opsin molecule. WT—wild-type.

It has been shown that p.G90D affects rhodopsin in a way that increases its constitutional activity [10,13,15,48,49]. This results in activation of the phototransduction cascade in the absence of light, thus desensitizing rod photoreceptors and leading to night blindness [50]. We hypothesize that vitamin A in its 11-*cis*-retinal form plays a role in stabilizing the constitutively active rhodopsin in patients with the p.G90D variant. Opsin becomes inactive after the binding of 11-*cis*-retinal [12,14]; therefore, the concentration of cis-retinal could be crucial in reducing the activity of constitutively active p.G90D opsin (Figure 5). Inactivation of opsin helps in shutting down the transduction cascade, reducing its toxicity and regaining light sensitivity. If this is the case, vitamin A may not be equally beneficial to all genetic subtypes of RP. Nevertheless, supplementation with vitamin A in patients with this pathogenic variant and comparisons with other pathogenic variants are needed to confirm our hypothesis. 

## 4. Materials and Methods

### 4.1. Patients

The study involved 29 patients from 7 families (13 female and 16 male) harboring p.G90D in *RHO*, with a median age of 44 years (range 8–73). One patient from each family (A:III-12, B:III-3, C:I-1, D:II-2, E:I-1, F: I-1 and G: I-1) was identified in the database of rare genetic eye diseases of the Eye Hospital of University Medical Centre Ljubljana, Slovenia. The other patients were recruited by inviting all family members who reported nyctalopia. The clinical features of 14 patients were described in a previous paper [17] (marked with * in Table 1), while 15 were identified afterwards.

All investigations were carried out in accordance with the Helsinki Declaration on Biomedical Research in Human Beings. The study was approved by the Commission of the Republic of Slovenia for Medical Ethics (Protocol 0120-435/2020/3, 20 October 2020). Patients signed informed consent.

### 4.2. Genetic and Bioinformatic Analysis

Genomic DNA was extracted from the blood samples according to the standard procedure. Genetic analysis was performed by whole exome sequencing in one proband from each family (A:III-12, B:III-3, C:II-2, D:II-2?, E:I-1, F:I-1, and G:I-1). Sequencing of the defined targets was performed using next-generation sequencing on the isolated DNA sample. Briefly, fragmentation and enrichment of the isolated DNA sample were performed according to the Illumina Nextera Coding Exome capture protocol, with subsequent sequencing on Illumina NextSeq 550 in 2 × 100 cycles. After the duplicates were removed, the alignment of reads to the UCSC hg19 reference assembly was performed using the Burrows–Wheeler aligner (BWA) algorithm (v0.6.3) and variant calling was performed using the GATK framework (v2.8). Only variants exceeding the quality score of 30.0 and depth of 5 were used for the down-stream analyses. Variant annotation was performed using ANNOVAR and snpEff algorithms, with pathogenicity predictions in the dbNSFPv2 database. Reference gene models and transcript sequences were based on the RefSeq database. Structural variants were assessed using the CONIFER v0.2.2 algorithm. Variants with a population frequency exceeding 1% in gnomAD, synonymous variants, intronic variants, and variants outside the clinical target were filtered out during the analyses. An in-house pipeline was used for the bioinformatic analyses of exome sequencing data, in accordance with the GATK best practice recommendations [51]. The interpretation of the sequence variants was based on the ACMG/AMP standards and guidelines [52]. When sequencing the DNA sample, we reached a median coverage of 67× and covered over 99.9% targeted regions with a minimum of 10× depth of coverage [53]. The presence of a pathogenic variant in other family members was confirmed using Sanger sequencing. Primers are available upon request.

### 4.3. Clinical Examination

Patients underwent a complete ophthalmological examination, which included Snellen visual acuity, color vision (Ishihara), slit lamp examination, perimetry (Goldmann perimetry using stimuli II/1 and II/4), color fundus photography (Topcon, Tokyo, Japan), FAF, optical coherence tomography (OCT; Spectralis, Heidelberg Engineering, Dossenheim, Germany), and ERG according to the standards and guidelines of International Society for Clinical Electrophysiology of Vision [54,55,56], using Espion (Diagnosys LLC, Lowell, MA, USA) or RETI scan (Roland Consult Stasche and Finger GmbH, Germany) visual electrophysiology testing systems. Wide-field color image and FAF were performed using Optos (Optos PLC, Dunfermline, UK) when possible. The FAF images were combined into mosaics using i2k Retina software (DualAlign LLC, Clifton Park, NY, USA). After scanning the visual fields, the area of the visual field for the individual isopters was determined using imageJ software (U.S. National Institutes of Health, Bethesda, MD, USA). 

### 4.4. Phenotype Classification

The phenotypes were classified into four categories, as described previously [17]: (1) night blindness without degeneration (NBWD), (2) Sector RP, (3) Pericentral RP, and (4) Classic RP. The characteristics of each phenotype are summarized in Table 3. For the purpose of this study, we decided to replace the term “congenital stationary night blindness (CSNB)”, commonly used to describe stationary night blindness without retinal degeneration, with a descriptive term “night blindness without degeneration (NBWD)”. The reasoning was that night blindness is not only a feature of CSNB, but also the first symptom of RP, and the progressive nature of the disease cannot be ruled out without long-term follow-up. 

### 4.5. Measurement of Serum Vitamin A Concentration

The concentration of vitamin A in the serum of patients was determined in the laboratory according to a standard protocol (SOP KIKKB 029/56 by University Medical Centre Ljubljana, Slovenia). In short, after the collection of a fasting blood sample in BD Vacutainer^®^ blood collection tubes with a red cap and no additives, the sample was protected from light by wrapping the tube in dark paper. With a brief sample preparation, the serum sample was purified from interfering substances. The method of choice for vitamin A determination was high-performance liquid chromatography (HPLC) (Thermo Fisher Scientific Inc., Waltham, MA, USA) with photometric detection. A light source with a wavelength of 325 nm was used. The emitted light was collected by the photocell of the detector. Vitamin A (*cis*-retinal) absorbs light at a wavelength of 325 nm, which is why it appeared as a peak on the chromatogram. The area of the peak was proportional to the concentration of vitamin A. For each sample (standard and control), a peak for vitamin A was identified. The program integrated the area of the peak and calculated the concentration of vitamin A with the help of external calibration, i.e., comparing the dot area of the sample with the dot area of the standard. The result was expressed in µmol/L. For the reference value, the laboratory reference values were used, namely 0.91–1.71 µmol/L (7–12 years old), 0.91–2.51 µmol/L (13–18 years old), and 1.05–2.8 µmol/L (adults).

### 4.6. Statistical Analysis

The statistical analysis was performed using IBM SPSS Statistics software for Windows (IBM Corp., Armonk, NY, USA). The median values were used to describe data with non-normal distributions. The association between non-normally distributed numerical variables was analyzed using Pearson correlation. The difference in the median values of the non-normally distributed variables was compared using the two-way Mann–Whitney U test (two groups) or the Kruskal–Wallis test (three or more groups). The association between the phenotype severity (mild vs. severe) and serum concentration of vitamin A, accounting for age, was analyzed using multiple logistic analysis. The value of *p* < 0.05 was used as the limit of statistical significance.

## 5. Conclusions

This study on a large group of patients with an identical p.G90D *RHO* variant showed a significant correlation between the serum concentration of vitamin A and the degree of retinal degeneration. Specifically, it appears that the vitamin A level plays a role in preventing retinal degeneration, and we hypothesize that the mechanism through which this occurs is stabilization of the constitutively active p.G90D rhodopsin at a higher concentration of 11-*cis*-retinal. The results are informative for potential clinical trials that would re-evaluate vitamin A in specific genetic types of RP.

## Figures and Tables

**Table 1:** Clinical characteristics of the included patients and their serum concentrations of vitamin A.

Patient ID (Centre ID)	Age When Examined (Years)	Sex	Phenotype	Serum Concentration of Vitamin A (µmol/L)	Normal Range of Serum Concentration of Vitamin A for Age (µmol/L)
A:III-10 * (642)	71	M	Classic RP	1.68	1.05–2.8
A:III-11 * (645)	65	M	Sector RP	2.93	1.05–2.8
A:III-12 * (604)	63	F	Sector RP	2.44	1.05–2.8
A:III-13 (1031)	54	M	Sector RP	3.04	1.05–2.8
A:III-14 * (760)	55	M	Sector RP	3.56	1.05–2.8
A:III-17 * (653)	65	M	Classic RP	2.74	1.05–2.8
A:IV-1 * (117)	48	M	NBWD	3.23	1.05–2.8
A:IV-4 * (1030)	39	M	Classic RP	2.42	1.05–2.8
A:IV-5 * (644)	36	M	Classic RP	2.48	1.05–2.8
A:IV-8 (1033)	30	F	NBWD	2.01	1.05–2.8
A:IV-17 * (652)	38	F	Classic RP	2.1	1.05–2.8
A:IV-19 (1034)	39	F	NBWD	1.11	1.05–2.8
A:V-1 * (1032)	17	M	NBWD	1.44	0.91–2.51
A:V-4 (1030)	10	F	Classic RP	0.89	0.91–1.71
B:III-3 * (610)	29	M	Pericentral RP	2.33	1.05–2.8
C:I-1 * (580)	66	F	Classic RP	2.34	1,05–2,8
C:II-2 * (612)	40	M	Classic RP	1.56	1.05–2.8
C:III-2 * (874)	8	M	NBWD	1.64	0.91–1.71
D:II-1 (1020)	73	M	Classic RP	2.47	1.05–2.8
D:II-2 (1021)	65	F	Classic RP	2.32	1.05–2.8
D:II-3 (1974)	59	M	Classic RP	1.78	1.05–2.8
D:III-1 (951)	44	M	Classic RP	2.62	1.05–2.8
D:IV-1 (950)	14	F	NBWD	1.95	0.91–2.51
E:I-1 (658)	57	F	Classic RP	1.53	1.05–2.8
E:I-2 (431)	63	M	Classic RP	1.23	1.05–2.8
E:II-1 (1058)	34	F	Classic RP	*0.92*	1.05–2.8
E:II-2 (1067)	23	M	Classic RP	1.38	1.05–2.8
E:II-3 (1068)	13	F	NBWD	1.32	0.91–2.51
F:I-1 (1961)	48	F	Classic RP	1.75	1.05–2.8
G:I-1 (1049)	56	F	Classic RP	2.25 (s)	1.05–2.8

M—male; F—female; RP—retinitis pigmentosa; NBWD—night blindness without degeneration; (s)—after one year of vitamin A supplementation. Serum concentrations of vitamin A above the age normative are written in bold and those below are written in italic. Patients whose detailed clinical picture was described in a previous paper (N = 14) [17] are marked with *. The laboratory reference values are 0.91–1.71 µmol/L (7–12 years old), 0.91–2.51 µmol/L (13–18 years old), and 1.05–2.8 µmol/L (adults).

**Table 2:** Multiple logistic regression on the effect of age and serum concentration of vitamin A on the phenotype severity.

	B	S.E.	Wald	df	Sig.	Exp(B)
Serum concentration of vitamin A	−1.730	0.839	4.249	1	0.039 *	0.177
Age	0.065	0.030	4.759	1	0.029 *	1.068
Constant	1.367	1.434	0.908	1	0.353	3.923

Mild phenotype (NBWD or Sector RP) was denominated with 0 and severe phenotype (Classic RP or Pericentral RP) with 1. * Significance below 0.05. B—logistic regression coefficient; S.E.—standard error; Wals—rating scale; df—degree of freedom; Sig.—statistical significance of the test; Exp(B)—exponentiation of the B coefficient.

**Table 3:** Features of the four phenotypic categories.

Phenotype	Main Features	Auxiliary Features
NBWD	Night blindness, normal visual field, normal fundus, normal FAF	Decreased or absent DA responses on ERG and normal LA responses on ERG
Sector RP	Night blindness, peripheral hypoautofluorescence on AF surrounded by a hyperautofluorescent line in the region of one or two quadrants	Partially constricted visual field, bone spicules in one or two quadrants, decreased DA and LA responses on ERG
Pericentral RP	Night blindness, annular area of retinal degeneration encompassed by a double hyperautofluorescent ring on FAF	Pericentral scotoma, retinal changes along the vascular arches
Classic RP	Night blindness, constricted visual field, concentric retinal degeneration delineated by a hyperautofluorescent ring on FAF	Bone spicules in three or four quadrants, decreased or absent DA and LA responses on the ERG

NBWD—night blindness without degeneration; RP—retinitis pigmentosa; FAF—fundus autofluorescence; DA—dark adapted; LA—light adapted; ERG—electroretinography.

## Data Availability

The original data are available upon reasonable request to the corresponding author.

## Abbreviations

RHO	Rhodopsin gene
RP	Retinitis pigmentosa
NBWD	Night blindness without degeneration
CSNB	Congenital stationary night blindness
GCPR	G-protein coupled receptor
FAF	Fundus autofluorscence imaging
RE	Right eye
LE	Left eye
ERG	Electroretinography
RPE	Retinal pigment epithelium
RBP	Retinal binding protein
STRA6	Retinoic acid 6
CRBP1	Cellular retinol -binding protein 1
LRAT	Retinol:lecithin acyltransferase
RPE65	Retinoid isomerohydrolase
CRALBP	Cellular retinaldehyde-binding protein
IRBP	Interphotoreceptor retinoid-binding protein
IU	International Unit
WT	Wild-type
DNA	Deoxyribonucleic acid
BWA	Burrows-Wheeler aligner
GATK	Genome analysis toolkit
CONIFER	Copy number inference from exome reads
GnomAD	Genome aggregation database
ACMG/AMP	American College of Medical Genetics and Genomics/Association for Molecular Pathology
OCT	Optical coherence tomography
DA	Dark adapted
LA	Light adapted
HPLC	High-performance liquid chromatography
